# Echocardiography and extravascular lung water during 3 weeks of exposure to high altitude in otherwise healthy asthmatics

**DOI:** 10.3389/fphys.2023.1214887

**Published:** 2023-07-25

**Authors:** S. Saxer, P. R. Bader, S. R. Schneider, M. Mademilov, U. Sheraliev, P. Appenzeller, J. Müller, T. M. Sooronbaev, K. E. Bloch, S. Ulrich, M. Lichtblau

**Affiliations:** ^1^ Department of Pulmonology, University Hospital of Zurich, Zurich, Switzerland; ^2^ Swiss-Kyrgyz High Altitude Medicine and Research Initiative, Tuja-Ashu, Kyrgyzstan; ^3^ Eastern University of Applied Sciences, St Gallen, Switzerland; ^4^ Department of Respiratory Medicine, National Center for Cardiology and Internal Medicine, Bishkek, Kyrgyzstan

**Keywords:** hypoxia, altitude, echocardiography, asthma, acclimatization

## Abstract

**Background:** Asthma rehabilitation at high altitude is common. Little is known about the acute and subacute cardiopulmonary acclimatization to high altitude in middle-aged asthmatics without other comorbidities.

**Methods:** In this prospective study in lowlander subjects with mostly mild asthma who revealed an asthma control questionnaire score >0.75 and participated in a three-week rehabilitation program, we assessed systolic pulmonary artery pressure (sPAP), cardiac function, and extravascular lung water (EVLW) at 760 m (baseline) by Doppler-echocardiography and on the second (acute) and last day (subacute) at a high altitude clinic in Kyrgyzstan (3100 m).

**Results:** The study included 22 patients (eight male) with a mean age of 44.3 ± 12.4 years, body mass index of 25.8 ± 4.7 kg/m^2^, a forced expiratory volume in 1 s of 92% ± 19% predicted (post-bronchodilator), and partially uncontrolled asthma. sPAP increased from 21.8 mmHg by mean difference by 7.5 [95% confidence interval 3.9 to 10.5] mmHg (*p* < 0.001) during acute exposure and by 4.8 [1.0 to 8.6] mmHg (*p* = 0.014) during subacute exposure. The right-ventricular-to-pulmonary-artery coupling expressed by TAPSE/sPAP decreased from 1.1 by −0.2 [−0.3 to −0.1] mm/mmHg (*p* < 0.001) during acute exposure and by −0.2 [−0.3 to −0.1] mm/mmHg (*p* = 0.002) during subacute exposure, accordingly. EVLW significantly increased from baseline (1.3 ± 1.8) to acute hypoxia (5.5 ± 3.5, *p* < 0.001) but showed no difference after 3 weeks (2.0 ± 1.8).

**Conclusion:** In otherwise healthy asthmatics, acute exposure to hypoxia at high altitude increases pulmonary artery pressure (PAP) and EVLW. During subacute exposure, PAP remains increased, but EVLW returns to baseline values, suggesting compensatory mechanisms that contribute to EVLW homeostasis during acclimatization.

## Introduction

Worldwide, an estimated 300 million people are affected by asthma, with a varying prevalence of 1%–25% (Gourgoulianis et al., 2001; Droma et al., 2007; Global initiative for Asthma, 2023). Very little is known about pulmonary vascular and cardiac acclimatization in asthmatic but otherwise healthy middle-aged lowlanders traveling to and staying at high altitude (Cogo and Fiorenzano, 2009). Due to the potentially favorable effects of the altitude climate, many asthmatics are sent to high altitude for asthma rehabilitation in order to improve symptoms and lung function (Fieten et al., 2022). The fall in barometric pressure and decreased partial pressure of oxygen (PaO_2_) at high altitude cause compensatory increased minute ventilation and thus lead to hypocapnia as well as hypoxic pulmonary vasoconstriction (HPV). The resulting elevated pulmonary artery pressure (PAP) while traveling to high altitude has been associated with right-sided cardiac dysfunction and may predispose the patient to high-altitude pulmonary edema (HAPE), which is a life-threatening condition associated with increasing extravascular lung water (EVLW, B-lines) (Allemann et al., 2000; Maggiorini et al., 2001; Mounier et al., 2011; Swenson and Bartsch, 2012). When the severity of asthma is increased and during exacerbations, patients may develop hyperinflation that may challenge the cardiopulmonary system. Thus, the known increase in PAP while traveling to high altitude might increase the risk of right heart decompensation for these patients (Eniseeva and Sizykh, 1995; Bobrov et al., 2003).

Previous studies on the PAP and right heart function at high altitude have focused mainly either on acute short-term exposure in healthy younger travelers or highlanders permanently living at high altitude (Soria et al., 2016; Soria et al., 2019). However, studies investigating the subacute acclimatization of the right heart function are rare but are of particular interest for people traveling to high altitudes during vacations and for asthma patients undergoing high-altitude climate therapy (Fieten et al., 2022). Rehabilitation is an add-on of a non-pharmacological intervention in patients with asthma (Global Initiative for Asthma, 2023).

Therefore, the purpose of the current study was to describe the cardiac function and hemodynamics and perform sonographic assessments of EVLW in otherwise healthy asthmatic lowlanders staying for 3 weeks at a high altitude (3100 m) while participating in a comprehensive rehabilitation program.

## Materials and methods

### Design

This prospective study was embedded in a randomized controlled trial investigating the effect of 3 weeks of asthma rehabilitation at a high altitude (Saxer et al., 2019). We conducted three sequential measurements of PAP and cardiac function by echocardiography and lung sonography in patients with asthma. The first measurement was made at a low altitude baseline in Bishkek (Kyrgyzstan, altitude 760 m), and the second was made the day after arrival at the Too Ashu high-altitude clinic (Kyrgyzstan, altitude 3,100 m) by minibus (acute exposure), and the last was made after 17 nights at 3100 m (subacute hypoxia).

The patients participated in a rehabilitation program during the high-altitude stay, including patient education, endurance training, muscle strength training, breathing exercises, and guided walks. A detailed description of the intervention can be found in the previous publication (Saxer et al., 2019).

The study was performed between June and July 2016 and was approved by the Ethics Committee of the National Center of Cardiology and Internal Medicine, Bishkek, Kyrgyzstan (No. 01-8/151). All patients provided written informed consent.

## Patients

Patients with mild to moderate, atopic, or non-atopic asthma that was not optimally controlled were included in this current trial. All patients had lived in or near Bishkek below 1,000 m for the last 3 months. Patients with an underlying cardiac pathology, assessed by taking a history and echocardiography, were excluded; for details, see Saxer et al. (2019).

### Measurements and procedures

Resting transthoracic echocardiography was conducted by three experienced cardiac sonographers (P.B., M.L., and S.U.). The images were obtained with a CX 50 Ultrasound System (Philips^®^, Bothell, United States of America), using a 5 to 1 MHz sector array transducer, and analyzed after the examination on the same system by the same physicians. A standard echocardiographic investigation was performed according to guidelines (Quiñones et al., 2002; Rudski et al., 2010; Lang et al., 2015).

The tricuspid regurgitation pressure gradient (TRPG) was derived from the maximal tricuspid regurgitation jet velocity (TRVmax) using the modified Bernoulli equation. The systolic PAP (sPAP) was calculated by adding the maximal TRPG to the right atrial pressure (RAP), which was rated as 3, 8, or 15 mmHg, depending on the diameter and collapsibility of the inferior vena cava (Rudski et al., 2010; Lichtblau et al., 2019). The mean PAP (mPAP) was estimated by the equation mPAP = sPAP * 0.61 + 2 (Chemla et al., 2004).

The left ventricular systolic function was assessed by the ejection fraction (EF) according to the modified Simpson’s rule. Stroke volume (SV) was calculated using the velocity time integral and the diameter of the left ventricular outflow tract (LVOT). Cardiac output (CO) was derived by multiplying the SV by the heart rate (HR). Stroke volume index (SVI) and cardiac index (CI) were obtained by dividing SV and CO by the body surface area (BSA). The assessment of the right ventricular function included the following parameters: tricuspid annular plane systolic excursion (TAPSE), tricuspid annular systolic velocity (TASV), RV end-diastolic area (RV-EDA), RV end-systolic area (RV-ESA), right ventricular fractional area change (RV-FAC), diastolic right and left ventricular internal diameters and their ratios (RVIDd/LVIDd) from an apical four-chamber view, and right atrial end-systolic area (RA-ESA). The total pulmonary resistance was calculated with mPAP/CO, the right ventricular arterial coupling was calculated with TAPSE/sPAP (Tello et al., 2019), the pulmonary arterial wedge pressure (PAWP) was calculated with the formula (PAWP = 1.24 × (E/e’) + 1.9) (Nagueh et al., 2016), and the pulmonary vascular resistance (PVR) was calculated as with PVR = (mPAP − PAWP)/CO.

### Sonographic assessment of the lung

After the echocardiographic examination, all patients were assessed for EVLW by looking for the number of B-lines in a supine position with the same ultrasound machine and transducer described previously (Lichtblau et al., 2021a). A B-line is defined as an echogenic linear signal with a narrow origin from the pleural line that crosses the image parallel to the sector arrays. These B-lines, observed at 28 different intercostal sites (on the left hemithorax from the second to the fourth and on the right from the second to the fifth intercostal spaces in the parasternal, midclavicular, anterior axillary, and midaxillary lines), were totaled (Picano et al., 2006).

### Other measurements

In addition, systemic blood pressure (BP), heart rate (HR) and pulse oximetry (SpO_2_) spirometry, and a 6-min walk test (6 MWT) were obtained in the same time frame (Saxer et al., 2019).

### Intervention

Patients performed a comprehensive 3-week, in-patient rehabilitation program at a high altitude that was described previously (Saxer et al., 2019).

### Statistical analysis

The analyses included all available measurements at different time points. The analysis was performed per protocol. Missing data were not imputed.

Data are presented as mean and ± SD. EVLW (B-lines) and echocardiographic and physiological parameters were analyzed in linear mixed models with different time points (baseline, acute, and subacute hypoxia as fixed effects and subjects as random intercept). The average marginal effects (mean difference) induced by time were extracted from these regression models and expressed as mean change with 95% confidence intervals. Model assumptions were tested by visual inspection of the homogeneity and normality of the residuals and the random effects (Kentucky–Ascombe and Q–Q plots). A *p*-value threshold of <0.05 or a confidence interval not including zero was considered to be statistically significant. All statistical analyses were performed using R Studio (version 1.0.153, R Studio Inc., San Francisco, United States).

## Results

**FIGURE 1:**
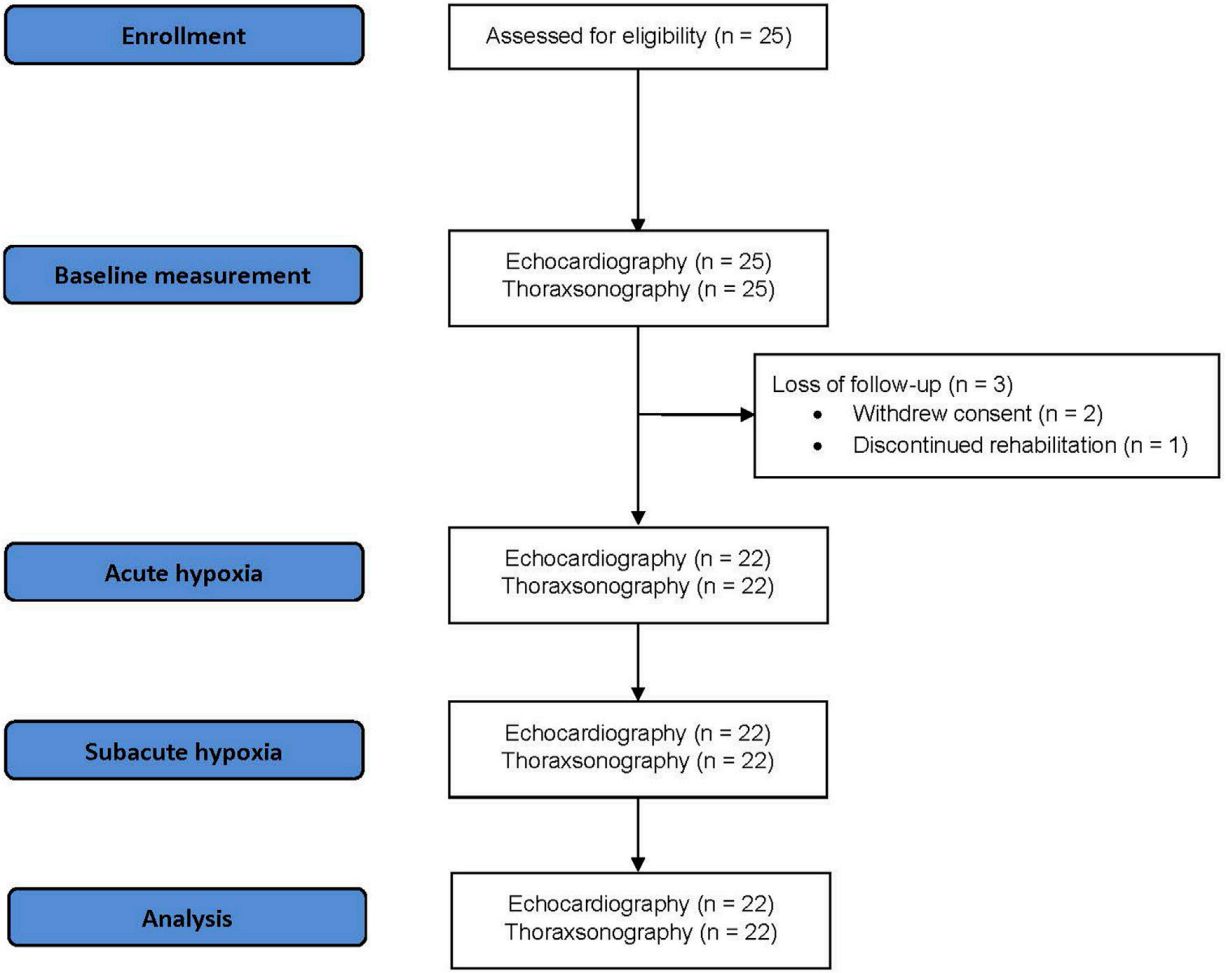
Patient flow.

Of 25 patients in the high altitude asthma rehabilitation study, 22 were included in this per-protocol study. Three patients were not available due to personal reasons (one discontinued the rehabilitation program, and two denied undergoing echocardiographic measures); see Figure 1.

Characteristics of the 22 patients (14 women), aged between 24 and 66 years, all non-smokers with no relevant cardiac comorbidity, are displayed in Table 1.

**TABLE 1:** Baseline characteristics at low altitude.

Subjects, n	22
Male	8 (36)
Female	14 (64)
Age, years	44.3 ± 12.4
Body mass index, kg/m^2^	25.8 ± 4.7
Body surface area, m^2^	1.78 ± 0.20
Height, m	1.66 ± 0.1
Spirometry pre-bronchodilator	
FEV1/FVC, %	63 ± 11
FEV1% predicted, %	80 ± 18
Spirometry post-bronchodilator	
FEV1% predicted, %	92 ± 19
Asthma severity by spirometry	
Mild (FEV1 predicted > 80%), n	20 (91)
Moderate (FEV1 predicted > 60%), n	2 (9)
Asthma Control Questionnaire score	2.33 ± 0.87
6-min walk distance, m	538 ± 58
Smokers, n	0 (0)
Medication	
Inhaled corticosteroids, n	22 (100)
Beta-adrenergics, n	17 (77)
Anticholinergics, n	1 (5)
Antihistamines, n	1 (5)
Leukotriene receptor antagonists, n	2 (9)

Data are presented as mean ± standard deviation or as number (%). SpO_2_, pulse oximetry; FEV1, forced expiratory volume in 1 s; FVC, forced vital capacity.

Table 2 displays the number of B-lines, oxygen saturation, and blood pressure. Almost all patients increased the number of B-lines in the acute exposure with a mean difference of 4.2 (2.7–5.7, *p* < 0.001) with a subsequent reduction over the course of the high-altitude stay and did not show a significant difference to the baseline (mean difference 0.7 [−0.8 to 2.2], *p* = 0.341). Oxygen saturation significantly decreased at altitude in the acute phase and only slightly recovered in the subacute phase (baseline: 95.5% ± 2.5% with a mean difference of −6.5 [−8.3 to −4.7]% during acute exposure and −4.1 [−5.9 to −2.3]% in the subacute exposure; all *p* < 0.001).

**TABLE 2:** Vital signs and extravascular lung water analyzed with a linear mixed model.

	Baseline, 760 m	Acute hypoxia, day 2 at 3,100 m	*p*-value	Subacute hypoxia at day 17 at 3,100 m	*p*-value
Heart rate, bpm	72.1 ± 10.9	74.5 ± 9.0	0.261	79.0 ± 9.1	0.001
Systolic blood pressure, mmHg	120 ± 24	117 ± 13	0.838	117 ± 11	0.410
Diastolic blood pressure, mmHg	76 ± 13	77 ± 12	0.708	80 ± 10	0.197
Oxygen saturation, %	95.5 ± 2.5	89.0 ± 5.4	<0.001	91.3 ± 2.7	<0.001
Weight, kg	71.0 ± 14.6	73.1 ± 13.2	0.070	73.0 ± 12.5	0.096
Lung comets, B-lines	1.3 ± 1.8	5.5 ± 3.5	<0.001	2.0 ± 1.8	0.341

Values are displayed as mean ± standard deviation. *p*-value linear mixed model compared to baseline.

There was no significant change in systolic and diastolic blood pressure (systolic: baseline 120 ± 24 to acute −3.2 [−10.6 to 4.2] mmHg to subacute −3.1 [−10.5 to 4.1] mmHg and diastolic: baseline 76 ± 13 to acute 1.0 [−5.0 to 6.0] mmHg to subacute 3.3 [−1.7 to 8.3] mmHg, all *p* > 0.05).

**FIGURE 2:**
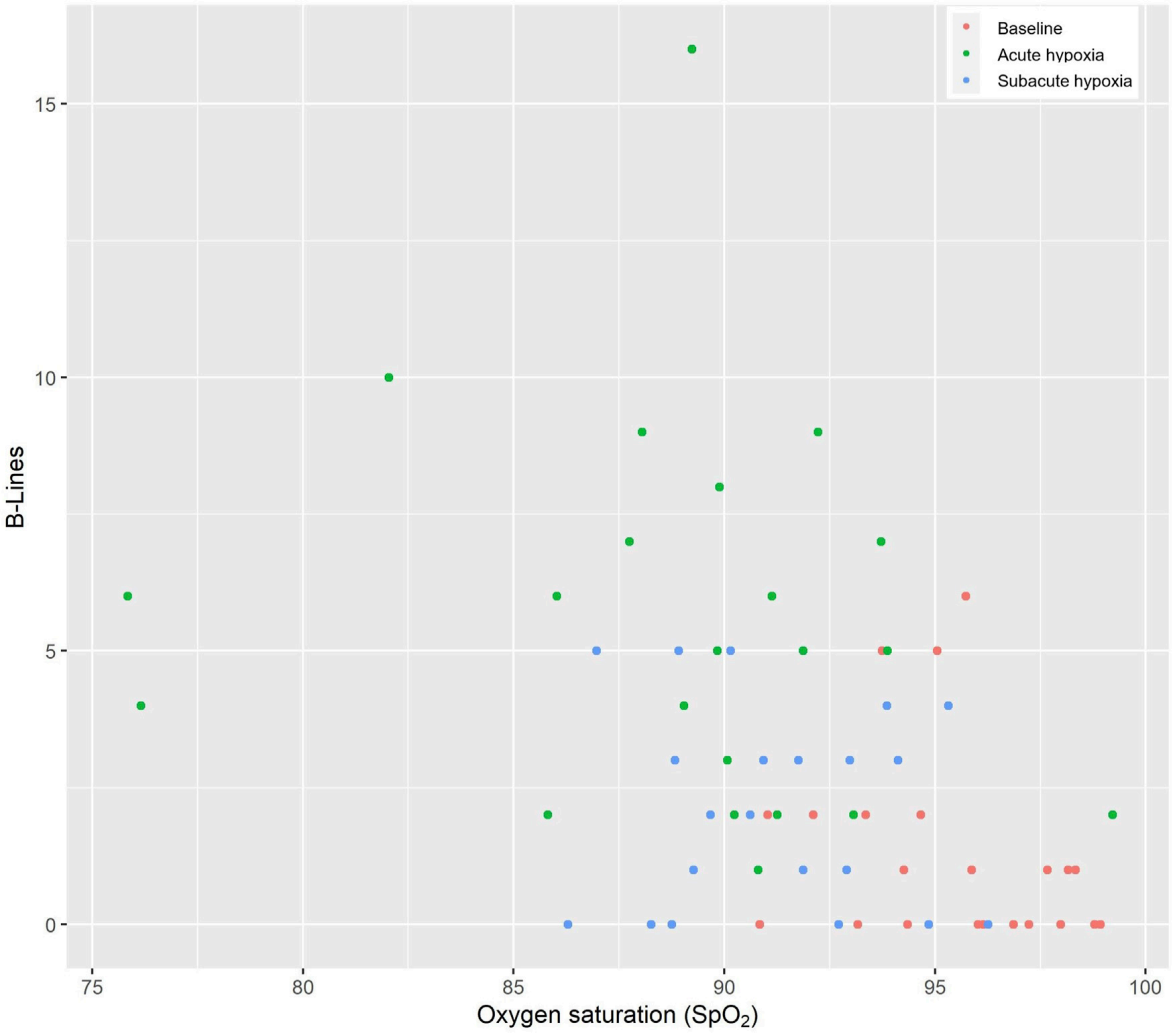
Thoraxsonography of the extravascular lung water (B-lines) and the oxygen saturation. The red circles indicate the baseline values at low altitude, the green circles indicate acute hypoxia, and the blue circles indicate subacute hypoxia.

Figure 2 displays the association of the EVLW and the SpO_2_ at the different measurement times −0.28 (−0.42 to −0.13, *p* < 0.001).

**FIGURE 3:**
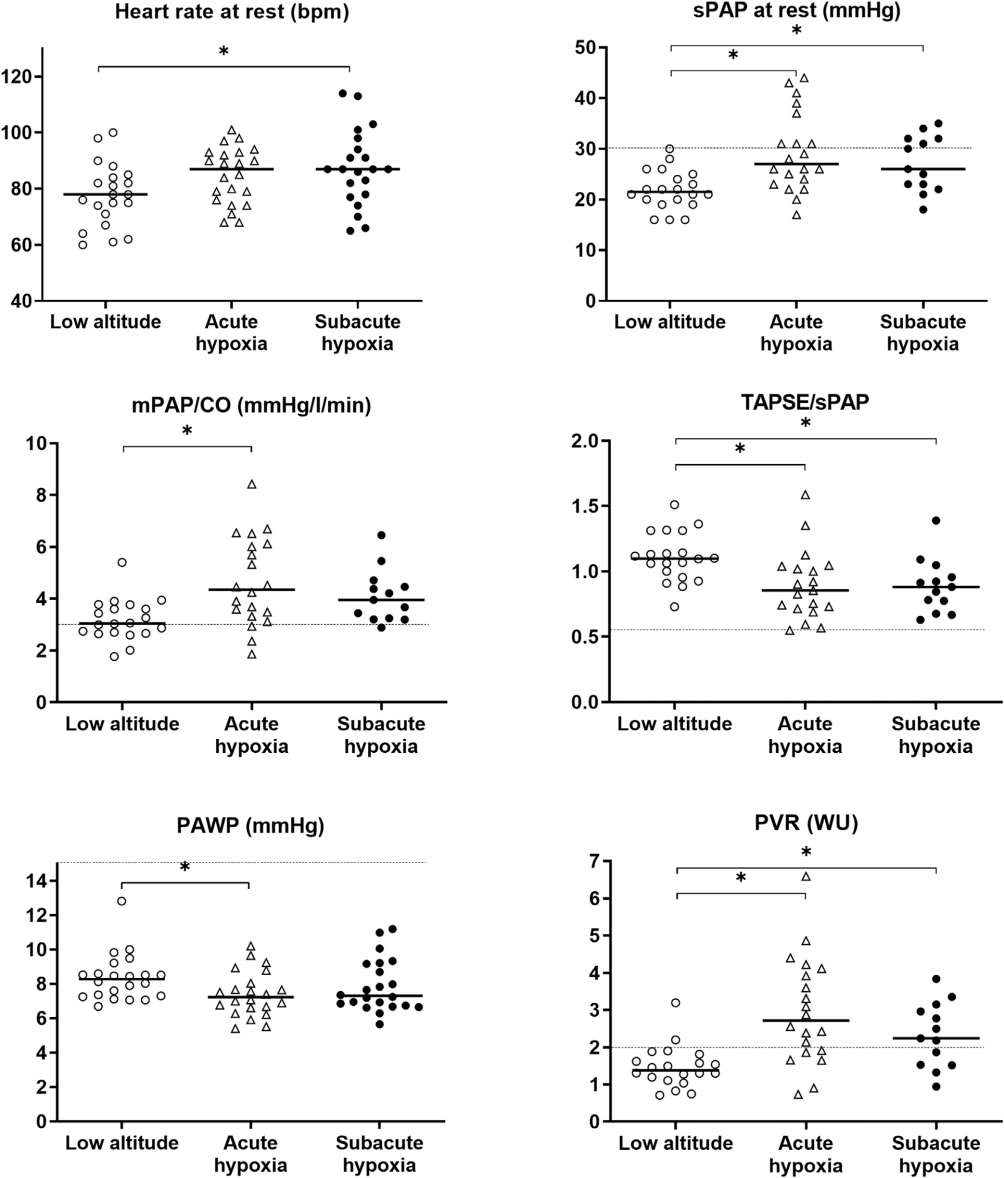
Grape plot of the mean pulmonary artery pressure (mPAP), the systolic PAP (sPAP), the total pulmonary resistance as the relationship of the cardiac output (mPAP/CO), the surrogate marker of right ventricular coupling (TAPSE/sPAP), the pulmonary arterial wedge pressure (PAWP), and the pulmonary vascular resistance (PVR). Empty circles display the individual values at baseline (low altitude), empty triangles indicate the values at high altitude 3,100 m (acute hypoxia), and full circles indicate the values at altitude with subacute exposure after 3 weeks. The dotted line shows the reference or cut-off values for healthy subjects under normobaric conditions (Humbert et al., 2022); the asterisks (*) mark the statistically significant differences.

The echocardiographic results are shown in Table 3 and Figure 3. Traveling to a high altitude revealed a significantly increased PAP and pulmonary vascular resistance as well as a reduced right-ventricular-to-pulmonary-artery coupling in acute hypoxia that was mainly maintained during subacute hypoxia.

**TABLE 3:** Echocardiographic parameters analyzed by a linear mixed model.

	Baseline, 760 m	Acute hypoxia, day 2 at 3,100 m	*p*-value	Subacute hypoxia at day 17 at 3,100 m	*p*-value
TRPG, mmHg	18.7 ± 3.7	25.1 ± 6.8	<0.001	22.4 ± 4.6	0.043
sPAP, mmHg	21.8 ± 3.9	29.2 ± 7.8	<0.001	27.1 ± 5.5	0.014
mPAP, mmHg	15.3 ± 2.4	19.8 ± 4.8	<0.001	18.5 ± 3.4	0.014
RAP, mmHg	3.2 ± 1.1	4.1 ± 2.1	0.081	4.1 ± 2.2	0.081
RA area, cm^2^	13.7 ± 2.1	13.3 ± 2.2	0.571	12.9 ± 2.6	0.210
RV FAC, %	42.1 ± 6.2	38.3 ± 8.2	0.071	35.6 ± 6.1	0.003
RV ESA, cm^2^	10.1 ± 2.6	11.8 ± 2.2	0.006	12.8 ± 2.8	<0.001
RV EDA, cm^2^	17.5 ± 4.5	19.3 ± 3.6	0.081	19.8 ± 4.0	0.028
TAPSE, cm	2.3 ± 0.3	2.4 ± 0.3	0.487	2.2 ± 0.2	0.101
TASV, cm/s	12.9 ± 1.9	12.8 ± 1.7	0.690	12.9 ± 1.7	0.947
SVI, mL/m^2^	38.5 ± 7.4	34.2 ± 7.1	0.009	34.6 ± 5.6	0.017
CI, L/min/m^2^	2.7 ± 0.5	2.5 ± 0.5	0.102	2.7 ± 0.4	0.806
mPAP/CO, mmHg/L/min	3.2 ± 0.8	4.6 ± 1.7	<0.001	4.1 ± 1.0	0.058
TAPSE/sPAP, mm/mmHg	1.1 ± 0.2	0.9 ± 0.3	<0.001	0.9 ± 0.2	0.002
TAPSE/TRPG, mm/mmHg	1.3 ± 0.2	1.0 ± 0.3	<0.001	1.1 ± 0.3	0.005
PAWP, mmHg	8.4 ± 1.4	7.4 ± 1.3	0.003	7.9 ± 1.5	0.155
PVR, WU	1.5 ± 0.6	3.0 ± 1.4	<0.001	2.3 ± 0.9	0.027
LFEF biplan, %	59.9 ± 4.8	61.0 ± 5.8	0.390	59.2 ± 4.4	0.582
LVID end-systolic, cm	3.0 ± 0.4	3.1 ± 0.5	0.138	3.0 ± 0.3	0.966
LVID end-diastolic, cm	4.5 ± 0.4	4.4 ± 0.5	0.182	4.3 ± 0.5	0.044
MV E/A	1.3 ± 0.5	1.0 ± 0.3	0.003	1.1 ± 0.3	0.014
Eccentricity index end-systolic	1.0 ± 0.1	1.0 ± 0.1	0.979	1.1 ± 0.1	0.033
Eccentricity index end-diastolic	1.1 ± 0.1	1.0 ± 0.1	0.297	1.1 ± 0.1	0.520
RV/LV, %	79 ± 15	78 ± 9	0.766	75 ± 9	0.206

Data are presented as mean ± SD. *p*-value linear mixed model compared to baseline. TRPG, tricuspid regurgitation pressure gradient; sPAP, systolic pulmonary artery pressure; mPAP, mean pulmonary artery pressure; RAP, right atrial pressure; RA, right atrium; RV, right ventricle; EDA, end-diastolic area; ESA, end-systolic area; FAC, fractional area change; TAPSE, tricuspid annular plane systolic excursion; TASV: tricuspid annular systolic velocity; SVI, stroke volume index; CI, cardiac index; mPAP/CO, total pulmonary resistance; PAWP, pulmonary arterial wedge pressure; PVR, pulmonary vascular resistance; LV, left ventricle; ID, internal dimension; MV, mitral valve; E/A, ratio of transmitral early diastolic to late diastolic velocity.

sPAP increased from 21.8 mmHg by 7.5 [3.9 to 10.5] mmHg in the acute exposure and by 4.8 [1.0 to 8.6] mmHg in the subacute exposure compared to baseline. Pulmonary vascular resistance increased from 1.5 WU at baseline by 1.5 [0.9 to 2.1] WU in the acute exposure and 0.8 [0.1 to 1.5] WU in the subacute exposure, and the right ventricular coupling expressed by TAPSE/sPAP decreased from 1.1 at baseline by −0.2 [−0.3 to −0.1] mm/mmHg in the acute exposure and −0.2 [−0.3 to −0.1] mm/mmHg in the subacute exposure; all values are statistically significant.

The FAC of the right ventricle was significantly reduced in the subacute phase, and the size of the right ventricle was significantly larger than the baseline.

Most patients tolerated the high altitude very well; only one patient suffered from asthma exacerbation, defined as a decline in PEF of >12%, and one patient, who suffered from AMS during the high-altitude stay, was treated with acetazolamide (Saxer et al., 2019).

## Discussion

In this study, we showed for the first time the acute and subacute effects of exposure to the hypobaric hypoxic environment on PAP, heart function, and EVLW in patients with mostly mild but partly uncontrolled asthma who participated in a three-week asthma rehabilitation program at high altitude (3100 m). We could show that in accordance with healthy subjects, sPAP increased with acute exposure to high altitude, albeit remaining below a level that would define pulmonary hypertension (PH), along with an increased PVR and a slightly lower TAPSE/sPAP ratio at high compared to low altitude but still within normal range. These changes went along with a decrease in SpO_2_ and an increase in HR with altitude and were maintained after 17 nights at high altitude. All changes, with the exception of the PVR, remained below a level considered pathological at low altitude. EVLW was assessed as B-lines increased upon acute exposure to the high altitude but returned to low altitude baseline values after 17 days, suggesting compensatory mechanisms that contribute to EVLW homeostasis during acclimatization.

### Oxygenation and hemodynamic changes

As expected, SpO_2_ decreased from 95% to 89% upon acute exposure to the high altitude and slightly rose during subacute exposure but remained decreased compared to the low altitude. Mean systemic blood pressure did not change with acute or subacute exposure to the high altitude. Previous studies have shown that blood pressure tends to be elevated only in the first 10–24 h and then normalizes over the next days at a moderate altitude (∼2500 m), but several weeks of acclimatization are needed to normalize the blood pressure at higher altitudes (Calbet et al., 2003; Parati et al., 2014; Sagoo et al., 2017).

In contrast to most reports, resting HR was only slightly but not significantly increased during acute exposure to the high altitude. This may be due to increased HR at the low altitude due to sympathetic activity caused by inhaled sympathomimetic bronchodilators with no further changes at the high altitude under maintained therapy (Huez et al., 2009; Gaur et al., 2021). As in other studies of acclimatization to high altitudes, the HR further increased after 17 nights at high altitude (Huez et al., 2009; Gaur et al., 2021). The acute effect of high altitudes on PAP and PVR due to hypoxic pulmonary vasoconstriction is well known (Grünig et al., 2000; Maggiorini and Leon-Velarde, 2003; Grunig et al., 2009) and is usually reversible after descent (Hilty et al., 2016). As previously described in healthy subjects and patients with lung diseases, PAP rose upon acute exposure to the high altitude in our asthma patients (Lichtblau et al., 2019; Lichtblau et al., 2021b; Schneider et al., 2022). However, sPAP remained below what would be considered pathological. At the end of the 3-week rehabilitation program, the PAP was still higher than at the low altitude, which is consistent with other reports (Lichtblau et al., 2021a; Gaur et al., 2021).

The stroke volume index was reduced in the acute and subacute phases at high compared to the low altitude, whereas the cardiac index remained stable due to the increase in the heart rate during the subacute phase.

In contrast to our study, Huez et al. (2009) showed a higher cardiac output in lowlanders during acute exposure and after acclimatization. They showed adaptive changes in the diastolic function of both ventricles; however, pulmonary vascular resistance was not directly calculated but was stated as being only moderately increased.

Gaur et al. (2021) studied young, healthy male Kyrgyz and Indian residents during a comparable length of stay at a higher altitude (4100 m). They showed comparable changes for HR, stroke volume, PAP, and PVR as shown in the investigated asthma cohort and thus may indicate that the presently investigated patients with mostly mild asthma could stay at a high altitude without exaggerated changes in pulmonary or systemic hemodynamics. The acute effect of high altitudes on PAP and PVR due to hypoxic pulmonary vasoconstriction is well known (Grünig et al., 2000; Maggiorini and Leon-Velarde, 2003; Grunig et al., 2009) and is reversible after descent (Hilty et al., 2016). In contrast to our study, the cardiac index increased at high altitude, probably due to a higher increase in heart rate. An increase in cardiac output during acute exposure was also found in patients with other obstructive lung diseases such as chronic obstructive pulmonary disease (Lichtblau et al., 2023).

In non-acclimatized Chinese Han lowlanders, Liu et al. (2022) showed that subacute exposure led to an enlargement of the right ventricle and a decrease of the diastolic right ventricular function, whereas the systolic function only decreased after long-term exposure to high altitudes (working at high altitudes for more than two decades). In our study, only the right ventricular end-systolic area was significantly enlarged at acute and subacute measurements at high altitude. Right ventricular systolic function was preserved; TAPSE did not significantly change during the high-altitude stay; FAC decreased slightly and was significantly lower in the subacute phase. We do not have a certain explanation for these changes; however, baseline examinations were performed during the summer at Bishkek with temperatures ranging around 45°C, and participants might have been dehydrated during the assessment leading to reduced intravascular and intracardiac fluid volumes, whereas the temperatures were significantly colder at the higher altitude. However, all values measured at high altitude were still within the normal range, and changes were slight and in accordance with those expected in healthy subjects (Gaur et al., 2021).

A study from Turkey focused on the long-term effect of migrating to higher altitudes in lowlanders. That study performed echocardiography within 48 h and 6 months after arrival at a moderate altitude (1890 m) but did not take baseline measurements at the low altitude. In contrast to the Chinese study, the Turkish study showed that RV diastolic function is altered upon long-term exposure to moderate altitudes, whereas systolic function was preserved (Arisoy et al., 2016).

### Extravascular lung water

B-lines can be used to measure extravascular lung water. High-altitude exposure can lead to acute mountain sickness and HAPE (Fagenholz et al., 2007). The increase in EVLW might be explained initially by elevated hydrostatic pressure that causes capillary leakage followed by extravasation of large-molecular-weight proteins and erythrocytes in the interstitial space, creating an increased osmotic gradient. This can then lead to pulmonary edema (Bartsch et al., 2005). The participants of this study did not show any clinical symptoms or signs of HAPE. Studies have shown that HAPE usually develops after 2–5 days upon exposure to high altitudes (Maggiorini, 2006). Similar to the study of Lichtblau et al. (2021a) that assessed healthy subjects at a low (720 m) and a high altitude (5050 m) in Chile, we have shown in this study that upon acute exposure to a high altitude, the number of B-lines initially increased and then decreased upon acclimatization. Our study showed acclimatization in terms of B-lines and also a moderate recovery of SpO_2_. This is consistent with compensatory mechanisms that assure adequate homeostasis of lung water during prolonged exposure to hypobaric hypoxia.

## Limitations

Because all patients underwent an asthma rehabilitation program during the high-altitude stay, a distinction between training and altitude effects cannot be made. However, it seems unlikely that asthma rehabilitation would have had a significant effect on PAP and heart function. Furthermore, with the background knowledge of similar changes found in acclimatization studies of healthy lowlanders without rehabilitation programs, it is well conceivable that changes seen here are due to the high-altitude exposure and subsequent acclimatization and not due to the asthma rehabilitation program, albeit the 3 weeks of asthma rehabilitation were associated with a significant increase in exercise capacity measured by the 6-minute walk distance and the 1 min sit-to-stand test and better asthma control. In addition to forced expiratory volume in 1 s, exhaled nitric oxide and hemoglobin were also significantly increased after the rehabilitation at the high altitude compared to the rehabilitation at the low altitude (Saxer et al., 2019).

All but two patients in this study had mild asthma but initially revealed an asthma control questionnaire score >0.75, which improved with the rehabilitation program, and all were otherwise healthy (Saxer et al., 2019). High-altitude-induced changes found in this cohort were in the range expected from studies of healthy subjects (Gaur et al., 2021; Liu et al., 2022); however, we cannot know whether they would apply to patients with more severe and less controlled asthma who wish to undertake rehabilitation programs at a high altitude.

There were many missing values of the TRPG at the last assessment in subacute hypoxia; whereas TRPG was assessable in 82% of subjects at baseline and even in 86% of subjects at acute high altitude measures, it was only present in 59% of subjects after 17 nights at the high altitude. The reason for the failing presence of the TRPG during subacute exposure to the high altitude is unclear but may be explained by hitherto unexplained acclimatization mechanisms. Echocardiography is associated with a potentially relevant intra- and interobserver variability, which was reduced by using the same echocardiographers throughout the study and double-checking the measurements. Because no blood gas analysis was carried out, we were not able to determine arterial oxygen content and oxygen delivery.

## Conclusion

In this first comprehensive assessment of the PAP, heart function, and EVLW upon acute and subacute exposure to a high altitude during asthma rehabilitation, we showed that in patients with mostly mild asthma, in accordance with findings in healthy subjects, PAP increases with altitude and remains elevated for up to 3 weeks, albeit with all changes being below pathological values proposed at a low altitude.

## Data Availability

The raw data supporting the conclusion of this article will be made available by the authors, without undue reservation.

## Abbreviations

ANOVA, analysis of variance, AT, acceleration time; BSA, body surface area; CI, cardiac index; CO, cardiac output; EF, ejection fraction; FEV1, forced expiratory volume in 1 s; HR, heart rate; LVOT, left ventricular outflow tract; RA, right atrial area; RAP, right atrial pressure; RV-EDA, right ventricular end-diastolic area; RV-ESA, right ventricular end-systolic area; RV-FAC, right ventricular fractional area change; RVIDd/LVIDd, right to left ventricular internal diastolic diameter ratio; sPAP, systolic pulmonary artery pressure; SV, stroke volume; SVI, stroke volume index; TAPSE, tricuspid annular plane systolic excursion; TASV, tricuspid annular systolic velocity; TRV, tricuspid regurgitation jet velocity; TRPG, tricuspid regurgitation pressure gradient; O₂, oxygen.
