# Corrigendum: Echocardiography and extravascular lung water during 3 weeks of exposure to high altitude in otherwise healthy asthmatics

**DOI:** 10.3389/fphys.2023.1280696

**Published:** 2023-08-30

**Authors:** S. Saxer, P. R. Bader, S. R. Schneider, M. Mademilov, U. Sheraliev, P. Appenzeller, J. Müller, T. M. Sooronbaev, K. E. Bloch, S. Ulrich, M. Lichtblau

**Affiliations:** ^1^ Department of Pulmonology, University Hospital of Zurich, Zurich, Switzerland; ^2^ Swiss-Kyrgyz High Altitude Medicine and Research Initiative, Tuja-Ashu, Kyrgyzstan; ^3^ Eastern University of Applied Sciences, St Gallen, Switzerland; ^4^ Department of Respiratory Medicine, National Center for Cardiology and Internal Medicine, Bishkek, Kyrgyzstan

**Keywords:** hypoxia, altitude, echocardiography, asthma, acclimatization

In the published article the **Funding** statement was incomplete. The grant number of the Swiss National Science Foundation was missing: “The study was funded by the Zurich Lung League and the Swiss National Science Foundation.” The correct Funding statement appears below.

“The study was funded by the Zurich Lung League and the Swiss National Science Foundation (IZK0Z3_168254)”.

The authors apologize for this error and state that this does not change the scientific conclusions of the article in any way. The original article has been updated.

